# Attitudes Toward Blockchain Technology in Managing Medical Information: Survey Study

**DOI:** 10.2196/15870

**Published:** 2019-12-09

**Authors:** Yong Sauk Hau, Jae Min Lee, Jaechan Park, Min Cheol Chang

**Affiliations:** 1 Department of Business Administration, School of Business, Yeungnam University Gyeongsan-si Republic of Korea; 2 Department of Pediatrics College of Medicine Yeungnam University Taegu Republic of Korea; 3 Department of Physical Medicine and Rehabilitation College of Medicine Yeungnam University Taegu Republic of Korea

**Keywords:** electronic health records, attitude, medical staff, patient, surveys and questionnaires

## Abstract

**Background:**

The recently developed blockchain technology uses a peer-to-peer network to distribute data to all participants for storage. This method enhances data safety, reliability, integrity, and transparency. To successfully introduce blockchain technology to medical data management, it is essential to obtain consent from medical doctors and patients.

**Objective:**

The aim of this study was to examine medical doctors’ and patients’ attitudes toward the use of blockchain technology and interpret the findings within the framework of expectancy theory.

**Methods:**

In this questionnaire survey, we examined medical doctors’ (n=90) and patients’ (n=90) attitudes toward the use of blockchain technology in the management and distribution of medical information. The questionnaire comprised 8 questions that assessed attitudes toward new means of managing and distributing medical information using blockchain technology. Responses were rated on a scale that ranged from 1 (very negative) to 7 (very positive).

**Results:**

Medical doctors (mean 3.7-5.0) reported significantly more negative attitudes than patients (mean 6.3-6.8). Furthermore, self-employed doctors reported more negative attitudes than employed doctors and university professors.

**Conclusions:**

To successfully introduce blockchain technology to medical data management, it is necessary to promote positive attitudes toward this technology among medical doctors, especially self-employed doctors.

## Introduction

### Background

In addition to an increase in life expectancy and use of health care services, medical advances have also led to an exponential growth in the amount of patient medical information available [1]. At present, different hospitals store and manage different fragments of a patient’s medical information. This method of managing medical information entails 4 disadvantages [1]. First, it makes it difficult for medical doctors to fully identify patients’ medical information. Second, as doctors do not have access to patients’ complete medical records, they may subject patients to redundant medical examinations that they have previously undergone in other hospitals. Third, when an interhospital transfer occurs, a patient or his/her guardian will be required to print the medical information on paper, transfer or copy imaging scans onto a CD, and submit them to another hospital. This process is not only inconvenient to the patient or guardian, but the process also carries the risk of omission of certain aspects of patient medical information. Finally, there is a risk that the information that is stored in the hospital may be hacked and leaked.

The recently developed blockchain technology differs from the conventional method of data storage in a central server or specific institution. Specifically, it uses a peer-to-peer network that distributes the data to all participants for storage; these users are responsible for hosting and managing the data [2,3]. This technology enhances data safety, reliability, integrity, and transparency [2,3]. Conventional centralized networks store data in a central server so that only a single central institution has access to the information. In contrast, blockchain-based distribution networks assign an account that contains distributable data to each user [4,5]. This renders hacking virtually impossible because anyone who wishes to modify the stored data will have to simultaneously hack into a vast number of user accounts [4,5]. Given this advantage, blockchain technology has been applied in diverse fields, such as finance, distribution, logistics, public services, and arts [6].

Recently, technology has gained a growing interest in the field of medicine [7-11]. Particularly, the use of blockchain technology in the transfer of medical data allows the patient rather than the hospital to own medical data. As a result, the patient rather than the hospital has control over medical data [1,4,7]. This enables patients to conveniently submit a complete set of medical data to any hospital upon transfer, and it helps physicians gain a better understanding of patients on the basis of the submitted data and plan suitable treatments [1,4,7]. In addition, it can reduce health care costs because patients are less likely to be subjected to redundant medical examinations when complete medical information is available. Moreover, it greatly reduces the risk of potential disclosure of patient medical information [1,4,7]. These advantages of blockchain technology are expected to have a positive influence on clinicians and patients.

### Objectives

However, the initial stages of introduction of blockchain technology to medical practice may involve challenges. Particularly, the introduction of blockchain technology to medical data management necessitates the fulfillment of 2 criteria: (1) it must solve technical problems, and (2) health care service providers (ie, medical doctors) and consumers (ie, patients) must consent to its use. According to expectancy theory [12], which is a widely used theory of human motivation in the field of psychology, medical doctors and patients are more likely to use blockchain technology when they hold positive attitudes toward it, and they are less likely to use it when they hold negative attitudes toward it. In this regard, medical doctors’ and patients’ attitudes are salient factors that must be examined in research studies on the application of blockchain technology to medical data management because a person’s attitude toward using a new information technology is indicative of their likelihood of using it [13]. However, the extant research on the introduction of blockchain technology to the health care sector has not shed any light on medical doctors’ and patients’ attitudes. Therefore, this study attempted to examine medical doctors’ and patients’ attitudes toward the use of blockchain technology and interpret the findings within the framework of expectancy theory [12].

## Methods

### Survey

The investigations were carried out following the rules of the Declaration of Helsinki of 1975. We conducted a questionnaire survey among practicing medical doctors who were between the ages of 30 and 49 years. Before the participants responded to the questionnaires, they viewed a video. The authors do not have any associations or relationships with the company that produced the video. In addition, a questionnaire survey was conducted among patients who were between the ages of 30 and 49 years. We think that the medical doctors under the age of 30 years are not experienced in the medical field enough to conduct this survey, and some doctors and patients over the age 49 years might find it difficult to understand blockchain technology. These participants were recruited from the lobby of a university hospital, and they provided consent before their participation in the survey.

The questionnaire comprised items that assessed attitudes toward new means of managing and distributing medical information using blockchain technology [1,14,15]. The questionnaire comprised the following items: (1) unlike traditional methods of medical data management, which bestow complete control over medical information to the hospital, blockchain technology allows a patient to choose the extent to which their medical information is stored, distributed, and managed; (2) blockchain technology delivers each aspect of a patient’s medical information to medical doctors; (3) as blockchain technology renders it impossible for one to hack medical information, it enhances the security of patient medical information; (4) blockchain technology prohibits anyone from revising a medical chart without patient consent once it has been created by a medical doctor; (5) blockchain technology allows patients to access information anywhere and at any time; (6) as blockchain technology allows hospitals to exchange medical information, patients do not have to print medical charts on paper, copy imaging scans onto a CD, and submit them to another hospital; (7) as blockchain technology reduces the likelihood of patients being subjected to redundant medical examinations, it lowers health care costs and reduces the time that patients spend at a hospital; and (8) blockchain technology makes it possible for one to use standardized medical big data to enhance the precision and personalization of medical treatments.

The items that were used to measure attitudes toward blockchain technology were adapted from a validated measure that has been used in previous studies, such as those that have been conducted by Venkatesh et al [13] and Min et al [16]. Responses to each item were recorded on a scale that ranged from 1 (*very negative*) to 7 (*very positive*). Higher scores were indicative of more positive attitudes, whereas lower scores were indicative of more negative attitudes.

The item that was used to gauge the levels of medical doctors’ and patients’ previous knowledge about blockchain technology was adapted from a validated measure in a similar previous study by Bettman and Park [17]. The level of previous knowledge about blockchain technology was gauged on a scale ranging from 1 (*do not know it at all*) to 7 (*know it very well*).

### Statistical Analysis

The collected data were analyzed using SPSS Version 24.0 (IBM Corporation). The data were analyzed in 2 steps. In the first step, the demographic characteristics and attitudes of medical doctors and patients were compared using the Mann-Whitney *U* and Chi-square tests. In the second step, Kruskal-Wallis and Mann-Whitney *U* tests were conducted to compare the demographic characteristics and attitudes of self-employed and employed doctors and university professors; the least significant difference test was used as a posthoc test. The level of statistical significance was specified as *P*<.05.

## Results

### Demographics

A total of 90 medical doctors from 16 different departments and 90 patients participated in this survey. Table 1 presents their demographic characteristics, and Table 2 summarizes the distribution of medical doctors across the 16 different departments that they represented.

The results of the first step of data analysis revealed no significant differences in the demographic characteristics of medical doctors and patients (Table 1).

**Table 1:** Significance of the difference in the demographic characteristics of medical doctors and patients.

Demographic characteristics	Medical doctors (n=90)	Patients (n=90)	*P* value
Age (years), 30s:40s, n	62:28	60:30	.75
Gender, male:female, n	62:28	60:30	.75
Level of prior knowledge^a^, mean (SD)	3.0 (1.4)	3.0 (1.5)	.69

^a^Level of prior knowledge about blockchain technology was rated on a scale that ranged from 1 (Do not know it at all) to 7 (Know it very well). Higher scores are indicative of a higher level of prior knowledge about blockchain technology.

**Table 2:** Distribution of medical doctors across the departments that they represented (N=90).

Department	Frequency, n (%)
Rehabilitation and physical medicine	27 (30)
Pediatrics	14 (16)
Neurosurgery	6 (7)
Anesthesiology	6 (7)
Family medicine	6 (7)
General practice	6 (7)
Internal medicine	5 (6)
Orthopedics	3 (3)
Dermatology	3 (3)
Plastic surgery	3 (3)
Obstetrics and gynecology	3 (3)
Diagnostic medicine	3 (3)
Psychiatry	2 (2)
General surgery	1 (1)
Radiology	1 (1)
Radiation oncology	1 (1)

### Result of Survey

The emergent means for all items that measured attitudes toward blockchain technology (ie, >3.5) were indicative of positive attitudes among both medical doctors and patients (Table 3).

**Table 3:** Significance of the difference in attitudes toward the use of blockchain technology in the management of medical information between medical doctors and patients.

Blockchain technology application	Medical doctors, mean (SD)	Patients, mean (SD)	*P* value^a^
(1) Unlike traditional methods of medical data management, which bestow complete control over medical information to the hospital, blockchain technology allows a patient to choose the extent to which their medical information is stored, distributed, and managed.	3.9 (1.9)	6.4 (0.8)	*<.001*
(2) Blockchain technology delivers each aspect of a patient’s medical information to medical doctors.	4.4 (1.9)	6.3 (0.9)	*<.001*
(3) As blockchain technology renders it impossible for one to hack medical information, it enhances the security of patient medical information.	4.4 (1.8)	6.8 (0.5)	*<.001*
(4) Blockchain technology prohibits anyone from revising a medical chart without patient consent once it has been created by a medical doctor.	3.7 (1.9)	6.4 (1.0)	*<.001*
(5) Blockchain technology allows patients to access information anywhere and at any time.	4.2 (1.9)	6.7 (0.6)	*<.001*
(6) As blockchain technology allows hospitals to exchange medical information, patients do not have to print medical charts on paper, copy imaging scans onto a CD, and submit them to another hospital.	5.0 (1.9)	6.8 (0.5)	*<.001*
(7) As blockchain technology reduces the likelihood of patients being subjected to redundant medical examinations, it lowers health care costs and reduces the time that patients spend at a hospital.	4.7 (2.0)	6.8 (0.5)	*<.001*
(8) Blockchain technology makes it possible for one to use standardized medical big data to enhance the precision and personalization of medical treatments.	4.7 (2.0)	6.7 (0.8)	*<.001*

^a^Values in italics are significant at the .05 level of significance.

However, medical doctors reported significantly more negative attitudes than patients across all items (mean 3.7-5.0; Table 3). Medical doctors reported the most negative attitude (mean 3.7) for the following item (ie, item 4): “blockchain technology prohibits anyone from revising a medical chart without patient consent once it has been created by a medical doctor.” The second most negative attitude (mean 3.9) was reported for the following item (ie, item 1): “unlike traditional methods of medical data management, which bestow complete control over medical information to the hospital, blockchain technology allows a patient to choose the extent to which their medical information is stored, distributed, and managed.” Meanwhile, patients reported more positive attitudes than medical doctors across all items (mean 6.3-6.8; Table 3).

The results of the second step of data analysis revealed no significant difference in the demographic characteristics of self-employed and employed doctors and university professors (Table 4).

Self-employed doctors reported significantly more negative attitudes than employed doctors and university professors on the following two items (Table 5): “since blockchain technology allows hospitals to exchange medical information, patients do not have to print medical charts on paper, copy imaging scans onto a CD, and submit them to another hospital” (item 6) and “since blockchain technology reduces the likelihood of patients being subjected to redundant medical examinations, it lowers health care costs and reduces the time that patients spend at a hospital.” (item 7). There was no significant difference among the 3 groups on the other items (Table 5).

**Table 4:** Significance of the difference in the demographic characteristics of self-employed and employed doctors and university professors.

Demographic characteristics	Self-employed doctors (n=14)	Employed doctors (n=54)	University professors (n=22)	*P* value
Age (years), 30s:40s, n	7:7	13:41	14:8	.15
Gender, male:female, n	12:2	36:18	14:8	.32
Level of prior knowledge^a^, mean (SD)	3.1 (1.5)	3.0 (1.5)	2.8 (1.4)	.74

^a^Level of prior knowledge about blockchain technology was rated on a scale that ranged from 1 (*Do not know it at all*) to 7 (*Know it very well*). Higher scores are indicative of a higher level of prior knowledge about blockchain technology.

**Table 5:** Significance of the difference in attitudes toward new methods of managing medical information using blockchain technology among self-employed doctors, employed doctors, and university professors.

Blockchain technology application	Self-employed doctors, mean (SD)	Employed doctors, mean (SD)	University professors, mean (SD)	*P* value
(1) Unlike traditional methods of medical data management, which bestow complete control over medical information to the hospital, blockchain technology allows a patient to choose the extent to which their medical information is stored, distributed, and managed.	3.0 (1.9)	4.0 (1.9)	4.4 (1.7)	.09
(2) Blockchain technology delivers each aspect of a patient’s medical information to medical doctors.	3.5 (2.4)	4.5 (1.9)	4.8 (1.6)	.10
(3) As blockchain technology renders it impossible for one to hack medical information, it enhances the security of patient medical information.	4.5 (2.0)	4.4 (1.8)	4.5 (1.8)	>.99
(4) Blockchain technology prohibits anyone from revising a medical chart without patient consent once it has been created by a medical doctor.	3.1 (1.9)	3.9 (2.1)	3.5 (1.5)	.42
(5) Blockchain technology allows patients to access information anywhere and at any time.	3.3 (2.1)	4.3(1.9)	4.7 (1.6)	.07
(6) As blockchain technology allows hospitals to exchange medical information, patients do not have to print medical charts on paper, copy imaging scans onto a CD, and submit them to another hospital.	3.5 (2.4)	5.2 (1.8)	5.4 (1.3)	*.02^a,b^*
(7) As blockchain technology reduces the likelihood of patients being subjected to redundant medical examinations, it lowers health care costs and reduces the time that patients spend at a hospital.	2.9 (2.4)	5.0 (1.9)	5.1 (1.7)	*.02^a,b^*
(8) Blockchain technology makes it possible for one to use standardized medical big data to enhance the precision and personalization of medical treatments.	3.8 (2.4)	4.9 (2.0)	5.0 (1.9)	.14

^a^Mean item score for self-employed doctors < mean item score for employed doctors and university professors.

^b^Values in italics are significant at the .05 level of significance.

## Discussion

### Principal Findings

This study examined medical doctors’ and patients’ attitudes toward new methods of managing and distributing medical information using blockchain technology. The major findings of this study can be summarized into 3 points. First, medical doctors reported significantly more negative attitudes than patients across all items that measured attitudes toward the application of blockchain technology to the management and distribution of medical information (Table 3). Second, self-employed doctors reported significantly more negative attitudes than employed doctors and university professors on the sixth and seventh items, which pertain to 2 advantages of blockchain technology, namely, convenient exchange of medical information among hospitals and lower number of redundant medical examinations (Table 5). Third, patients obtained means that ranged from 6 to 7 across all the items; this indicates that they held very positive attitudes toward the application of blockchain technology to medical data management (Table 3).

The 3 major findings of this study can be interpreted within the framework of the expectancy theory of psychological motivation [12], which suggests that people are motivated to pursue positive outcomes and avoid negative outcomes [12,18]. In this regard, the first finding can be interpreted to mean that medical doctors have more negative attitudes about blockchain technology than patients as they expect its use to lead to negative outcomes. For example, medical doctors’ attitudes were most negative for the fourth item, which delineates how blockchain technology makes it difficult for one to revise medical charts (Table 3). This feature of blockchain technology may be disadvantageous to medical doctors, when there is a medical dispute between them and a patient or guardian. Similarly, the second most negative attitudes were reported for the first item, which articulates the fact that blockchain technology allows patients to have complete control over their medical data (Table 3). This may have caused medical doctors to be concerned about potential misuse of patient medical information. The second major finding of this study has practical implications as it suggests that the use of blockchain technology may lead to negative outcomes among self-employed doctors. According to expectancy theory [12], there is a perceived association between a person’s actions and their consequent rewards [12,18]. Rewards may be of various types (eg, money and praise from other people) [12,18]. Self-employed doctors reported significantly more negative attitudes than employed doctors and university professors on the sixth and seventh items (Table 5). These items pertain to 2 advantages of blockchain technology: (1) it facilitates convenient transfer of medical information among hospitals, and (2) it lowers health care costs and reduces the time that patients spend at a hospital by minimizing the need for redundant medical examinations. As the profits that are earned from medical examinations contribute to self-employed doctors’ incomes, they may worry that the use of blockchain technology will lower their income. More specifically, patients may decline undergoing a medical examination that has been recommended by a smaller clinic if they have already undergone similar examinations in a larger hospital, even when such examinations are required to make an accurate medical diagnosis.

The third finding of this study suggests that patients hold very favorable attitudes toward the use of blockchain technology. This may be because of the fact that the application of blockchain technology to medical data management will yield outcomes that are beneficial rather than harmful to patients. Expectancy theory suggests that people cognitively analyze the gains and losses of behaviors in which they intend to engage [12,18]. In this regard, blockchain technology may offer patients several gains and lead to only a few losses. For instance, it grants patients direct control over their medical information and enhances the transparency and stability of their medical records [2,3]. Moreover, blockchain technology can decrease health care costs by diminishing the need for redundant medical examinations and making it easier to transfer patient medical information to another hospital [1]. In addition, it can contribute to medical research by granting researchers access to patients’ personal medical data [1,4].

The 3 major findings of this study, which are consistent with the premises of expectancy theory [12], suggest that to successfully introduce blockchain technology to medical data management, it is necessary to promote positive attitudes toward this technology among medical doctors. This can be achieved by alleviating their concerns about the adverse outcomes of the introduction of blockchain technology to medical data management and raising awareness about its potential advantages to medical doctors. In addition, the findings suggest that it is especially necessary to alleviate self-employed doctors’ concerns about potential problems that may result from introducing blockchain technology to medical data management.

At present, many companies worldwide are researching the means by which blockchain technology can be introduced to the health care sector [4,6]. Some pilot projects have been conducted in the domains of management and distribution of medical data. Although various studies have actively examined the new means by which blockchain technology can be applied to medical data management in the health care sector [2-7], none of the past studies have examined the attitudes of direct users of blockchain technology in health care sectors, namely, medical doctors and patients.

### Conclusions

In this study, we ascertained the extent to which the attitudes of medical doctors and patients are favorable toward the use of blockchain technology in the management and distribution of medical information. We found that medical doctors reported more negative attitudes than patients. Therefore, to introduce blockchain technology to medical data management, it is necessary to alleviate the concerns that medical doctors (especially self-employed doctors) have about the use of blockchain technology. Our study has some limitations. First, we did not examine the specific factors that lead to negative and positive attitudes toward the use of blockchain technology in medical data management. Second, diverse age groups were not represented in the study sample. Third, the number of doctors working in a specific department (physical medicine and rehabilitation) is disproportionately high. Fourth, the number of included subjects was relatively small. Fourth, a qualitative analysis method, such as a qualitative survey, can be effectively used to clearly show medical doctors’ and patients’ attitudes toward blockchain technology. However, we analyzed them by using only the quantitative analysis method using a questionnaire-based survey. Future studies that address these limitations will be able to enrich the present findings.

## Abbreviations

NRF: National Research Foundation
